# Molecular epidemiology of New Delhi metallo-β-lactamase-producing *Escherichia coli* in retail market chickens, Shandong, China

**DOI:** 10.3389/fmicb.2025.1550742

**Published:** 2025-04-22

**Authors:** Jing-Xian Ma, Shuan-Cheng Bai, Jia-Qi Xu, Zhao-Qing He, Yu-Xiang Qi, Jia-xin Wang, Yu-Xia Shi, Yu-Bao Li, Min-Ge Wang

**Affiliations:** ^1^College of Agriculture and Biology, Liaocheng University, Liaocheng, China; ^2^College of Smart Agriculture, Yulin Normal University, Yulin, China; ^3^College of Pharmaceutical Sciences and Food Engineering, Liaocheng University, Liaocheng, China

**Keywords:** carbapenemase, *Escherichia coli*, *bla*_NDM_, MLST, ARGs

## Abstract

**Background:**

The global spread of carbapenem-resistant *Escherichia coli* is a major public health concern. An investigation of their presence in the human and food chain products would facilitate the elucidation of the route of their food-borne transmission. Thus, the aim of this study was to investigate the prevalence of NDM-positive *E. coli* isolates in chicken at retail markets in Shandong, China.

**Methods:**

A total of 60 NDM-positive isolates were recovered from 531 *E. coli* isolates obtained from chickens at the retail market in Shandong. Antimicrobial susceptibility testing and polymerase chain reaction screening were performed to investigate the phenotype and genotype of carbapenemase resistance. Genomic characteristics of the -producing isolates were determined by WGS and bioinformatic analysis.

**Results:**

All of these isolates were multidrug-resistant (MDR), with a majority exhibiting resistance to meropenem, ampicillin, ceftazidime, cefotaxime, florfenicol, sulfamethoxazole/trimethoprim, and tetracycline. Whole genome sequencing (WGS) analysis indicated that these isolates were belonged to 18 distinct sequence types (STs), with the most prevalent STs being ST515 (17/60) and ST69 (11/60). Additionally, WGS analysis revealed that clonal spread of NDM-positive ST69 and ST515 *E. coli* isolates at markets in different cities in Shandong. Phylogenomic analysis showed that NDM-positive *E. coli* isolates from chickens were closely related to those of human origin.

**Conclusion:**

This study provides a new insight into the spread of NDM-positive *E. coli* isolates from retail chicken, and offers essential data for public health management.

## Introduction

The overuse of antibiotics can lead to the emergence of antimicrobial-resistant microorganisms in food-producing animals and in products derived from them, such as meat, eggs, and milk (Hassan et al., 2021; Chu et al., 2024; Sun et al., 2024). Consuming or coming into contact with food containing antimicrobial-resistant microorganisms can lead to the development of foodborne diseases that are challenging to treat, posing a significant threat to anti-infective treatment (Peng et al., 2024). Global annual deaths from foodborne illnesses caused by bacteria and other microorganisms exceed 400,000 (Todd, 2020). Therefore, the prevalence of carbapenem-resistant isolates is a public health concern that needs to be addressed on a global scale.

The main mechanism of carbapenem resistance is the production of carbapenemases. New Delhi metallo-β-lactamase (NDM) is one of the most common carbapenemases and has the strongest hydrolytic activity, hydrolyzing all β-lactam antibiotics except aztreonam (Kaewnirat et al., 2022). Since the discovery of NDM in clinical *Escherichia coli* in 2008, NDM-positive *E. coli* isolates have been shown to be globally distributed, with NDM-positive strains detected in virtually all countries conducting epidemiological surveys (Yong et al., 2009). To date, NDM-positive *E. coli* isolates have been recovered from hospitals and hospital sewage in major provinces/municipalities in China (Li et al., 2024; Liu et al., 2024). In addition, carbapenem antibiotics have never been approved for use in food-producing animals in the world; however, there have been sporadic reports of NDM-positive *E. coli* isolates from various food-producing animals and the downstream meat production chain (Bai et al., 2022; Wang et al., 2022; Wen et al., 2023). It is worth noting that NDM-positive *E. coli* isolates were highly prevalent among the Chinese poultry production chain, including commercial broiler farms, slaughterhouses, and supermarkets (Fu et al., 2024). In recent years, sporadic cases of NDM-positive *E. coli* isolates from chickens have been detected in retail markets in some local areas (Lv et al., 2022), suggesting that chickens in retail markets may be a “reservoir” for NDM-positive *E. coli* isolates. However, a large-scale survey of the NDM-positive *E. coli* isolates from retail market chickens remains unexplored.

In this study, we conducted a large-scale investigation on the prevalence of NDM-positive *E. coli* isolates in chickens from retail markets in Shandong, China. The antibiotic resistance phenotype and genotype, genetic diversity, phylogenetic relationships, and genetic environments of *bla*_NDM_ were evaluated. The findings of this study will provide fundamental data to help guide the development of food safety policies and ensure public health.

## Materials and methods

### Isolates collection

A total of 531 chicken samples were randomly collected from retail markets (5–8 chicken samples in each retail market) across 8 cities [Liaocheng (*n* = 75), Jinan (*n* = 93), Dezhou (*n* = 47), Taian (*n* = 52), Yantai (*n* = 87), Linyi (*n* = 74), Qingdao (*n* = 39), and Weifang (*n* = 64)] of Shandong Province from April 2018 to October 2020. In brief, all samples were added to 1 mL of lysogeny broth (LB) and incubated for 16–18 h at 37°C, followed by inoculation onto MacConkey plates for 12 h. Then, red clones were selected for identification using MALDI-TOF MS Axima^™^ (Shimadzu-Biotech Corp., Kyoto, Japan) and 16S rRNA sequencing. Finally, all the *E. coli* isolates were stored in 30% glycerol broth at −80°C.

All isolates were resuscitated on LB broth, and for 16–18 h at 37°C, followed by inoculation onto MacConkey plates containing 1.0 mg/L meropenem. Five major carbapenemase resistance genes (*bla*_KPC_, *bla*_NDM_, *bla*_IMP_, *bla*_OXA-48-like_, and *bla*_VIM_) were detected in carbapenem-resistant isolates using polymerase chain reaction (PCR) with previously described primers (Lv et al., 2022).

### Antimicrobial susceptibility testing

The MICs of 16 antibiotics (ampicillin, cefotaxime, ceftazidime, aztreonam, amikacin, gentamicin, ciprofloxacin, nalidixic acid, tetracycline, tigecycline, doxycycline, fosfomycin, sulfamethoxazole/trimethoprim, meropenem, colistin, and florfenicol) for all recovery isolates were determined using agar dilution and interpreted according to the Clinical and Laboratory Standards Institute (CLSI) guidelines (CLSI, 2020). The breakpoints of colistin and tigecycline for Enterobacteriaceae were interpreted according to the EUCAST criteria (European Committee on Antimicrobial Susceptibility Testing, 2019). *E. coli* ATCC 25922 served as a quality control strain for susceptibility testing.

### Conjugation assay testing

To determine the transferability of the resistance genes, streptomycin-resistant *E. coli* strain C600 was used as the recipient, and the conjugation assay was performed using a filter mating method. Transconjugants were selected using MacConkey agar plates containing 1.0 mg/L meropenem and 1,500 mg/L streptomycin.

### WGS and phylogenetic analysis

The genomic DNA of all NDM-positive isolates was subjected to 250 bp paired-end WGS using the Illumina MiSeq system (Illumina, San Diego, CA, United States), and the paired-end Illumina reads were assembled using SPAdes v3.6.2.18 MLST (Bankevich et al., 2012). Antibiotic resistance genes (ARGs) and plasmid replicon types were analyzed using the Center for Genomic Epidemiology server.^1^

The hosts and countries of 196 *E. coli* isolates were retrieved from the NCBI database,^2^ and the assembly genomes of the 196 isolates were downloaded from the NCBI database (as of September 2024). All assembly genomes were used for core-genome alignments to produce a phylogenetic tree using the Parsnp software of the Harvest suite (Todd et al., 2014). In this pipeline, bases that have likely undergone recombination are removed using PhiPack (Bruen et al., 2006), and only columns passing a set of filters based on these criteria are considered reliable core-genome SNPs. The final set of core-genome SNPs was submitted to FastTree 2 for reconstructing a maximum likelihood phylogenetic tree using default parameters (Poon et al., 2010). The VCF file of all variants identified by Parsnp was then used to determine pair-wise single-nucleotide variant distances between the core genomes of all strains. For the phylogenetic tree, a reference genome was randomly selected using the ‘-r!’ switch. The lineages of the phylogenetic tree were defined using rhierbaps version 6.0 (Cheng et al., 2013). The heat map was generated using R 3.3.2 (R Foundation for Statistical Computing) and was used to construct the tree that was visualized using FigTree v1.4.2 and iTOL v4 (Letunic and Bork, 2019). Plasmid maps were generated using the BRIG.

## Results

### Prevalence of carbapenemase-producing *Escherichia coli* isolates

**Figure 1:**
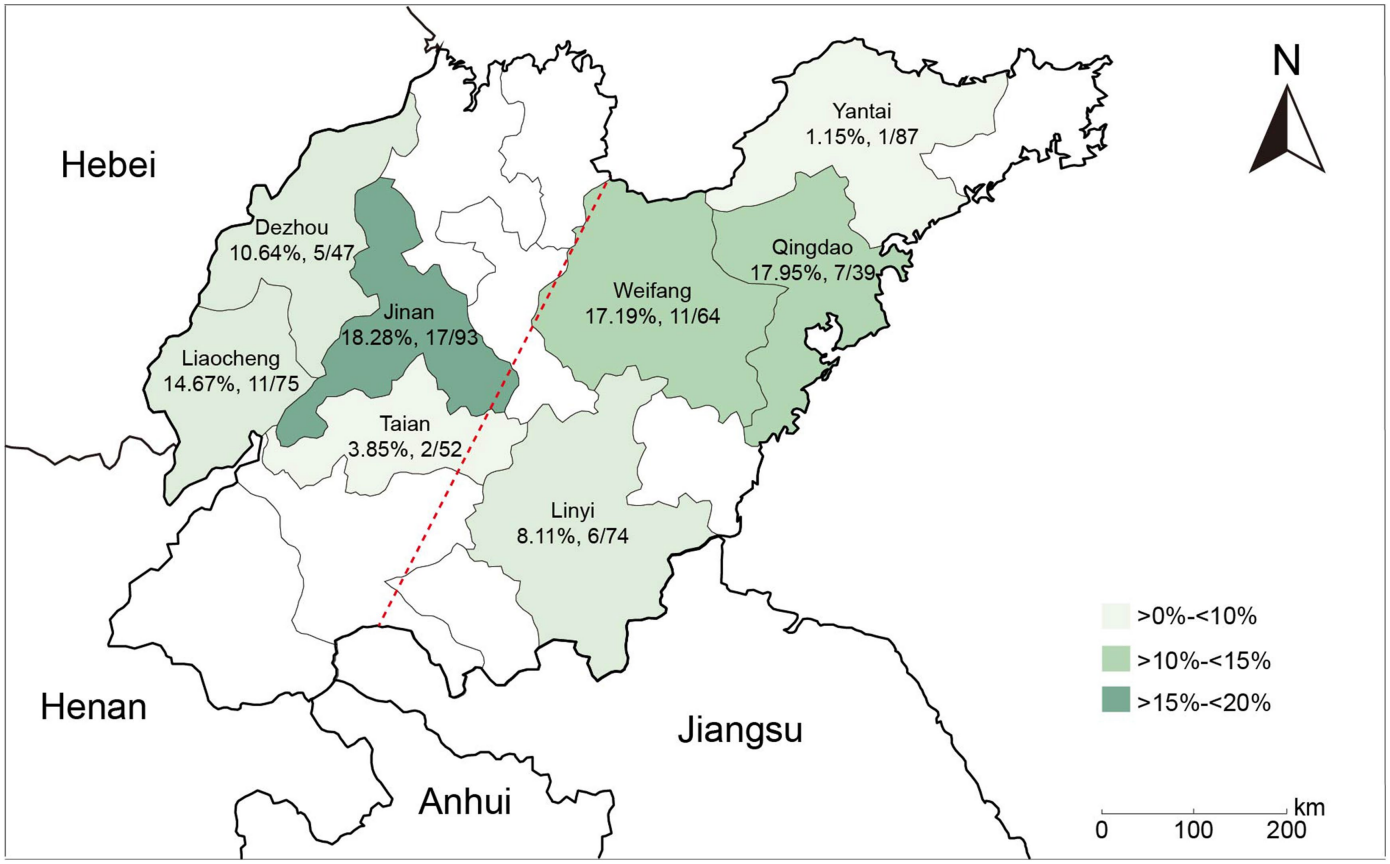
Geographical distribution of NDM-positive *E. coli* isolates in Shandong Province, China.

In this study, a total of 60 (11.30%) NDM-positive isolates were recovered from 531 *E. coli* isolates obtained from retail market chickens in Shandong, China. No other carbapenemase-encoding genes were detected among these carbapenem-resistant isolates. Among NDM-positive isolates, the most predominant variants were *bla*_NDM-5_ (86.67%, 52/60), followed by *bla*_NDM-9_ (10.00%, 6/60), *bla*_NDM-1_ (1.67%, 1/60), and *bla*_NDM-20_ (1.67%, 1/60) (Supplementary Figure S1a). The highest detection rate of NDM-positive *E. coli* isolates was observed in Jinan (18.28%, 17/93), followed by Qingdao (17.95%, 7/39), Weifang (17.19%, 11/64), and Liaocheng (14.67%, 11/75). In contrast, Taian (3.85%, 2/52) and Yantai (1.15%, 1/87) displayed the lowest detection rates (Figure 1). Moreover, the prevalence of NDM-positive *E. coli* isolates was higher in northwestern Shandong (13.11%, 35/267) than in southeastern Shandong (9.47%, 25/264) (Supplementary Figure S1b).

### Antibiotic resistance phenotypes

**Figure 2:**
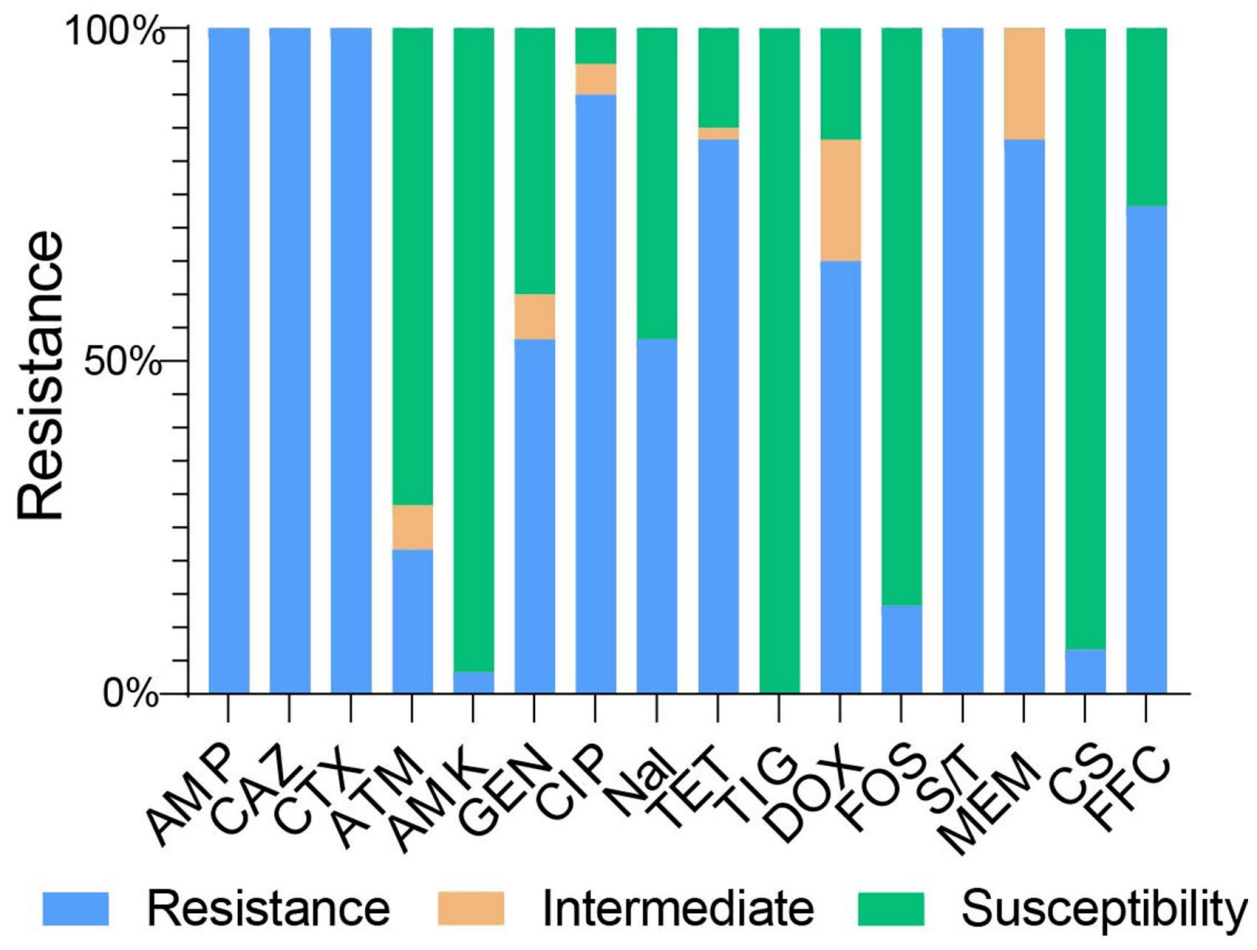
Minimum inhibitory concentrations of tested antimicrobial agents for the studied bacterial isolates. AMP, ampicillin; CTX, cefotaxime; CAZ, ceftazidime; ATM, aztreonam; AMK, amikacin; GEN, gentamicin; CIP, ciprofloxacin; Nal, Nalidixic acid; TET, tetracycline; TIG, tigecycline; DOX, doxycycline; FOS, fosfomycin; S/T, sulfamethoxazole/trimethoprim; MEM, meropenem; CS, colistin; FFC, florfenicol.

All 60 NDM-positive *E. coli* isolates exhibited resistance to ampicillin, cefotaxime, ceftazidime, and trimethoprim–sulfamethoxazole (Figure 2). In addition, the majority of these isolates remained resistant to meropenem (83.33%, 50/60), tetracycline (83.33%, 50/60), florfenicol (73.33%, 44/60), doxycycline (65.00%, 39/60), gentamicin (53.33%, 32/60), ciprofloxacin (90%, 54/60), and nalidixic acid (53.33%, 32/60). In contrast, a lower prevalence of resistance phenotypes was observed among aztreonam (21.67%, 13/60), amikacin (3.33%, 2/60), fosfomycin (13.33%, 8/60), and colistin (6.67%, 4/60) (Figure 2). Of note, none of the isolates showed resistance to tigecycline.

### Phylogenetic analysis of NDM-positive *Escherichia coli* isolates

**Figure 3:**
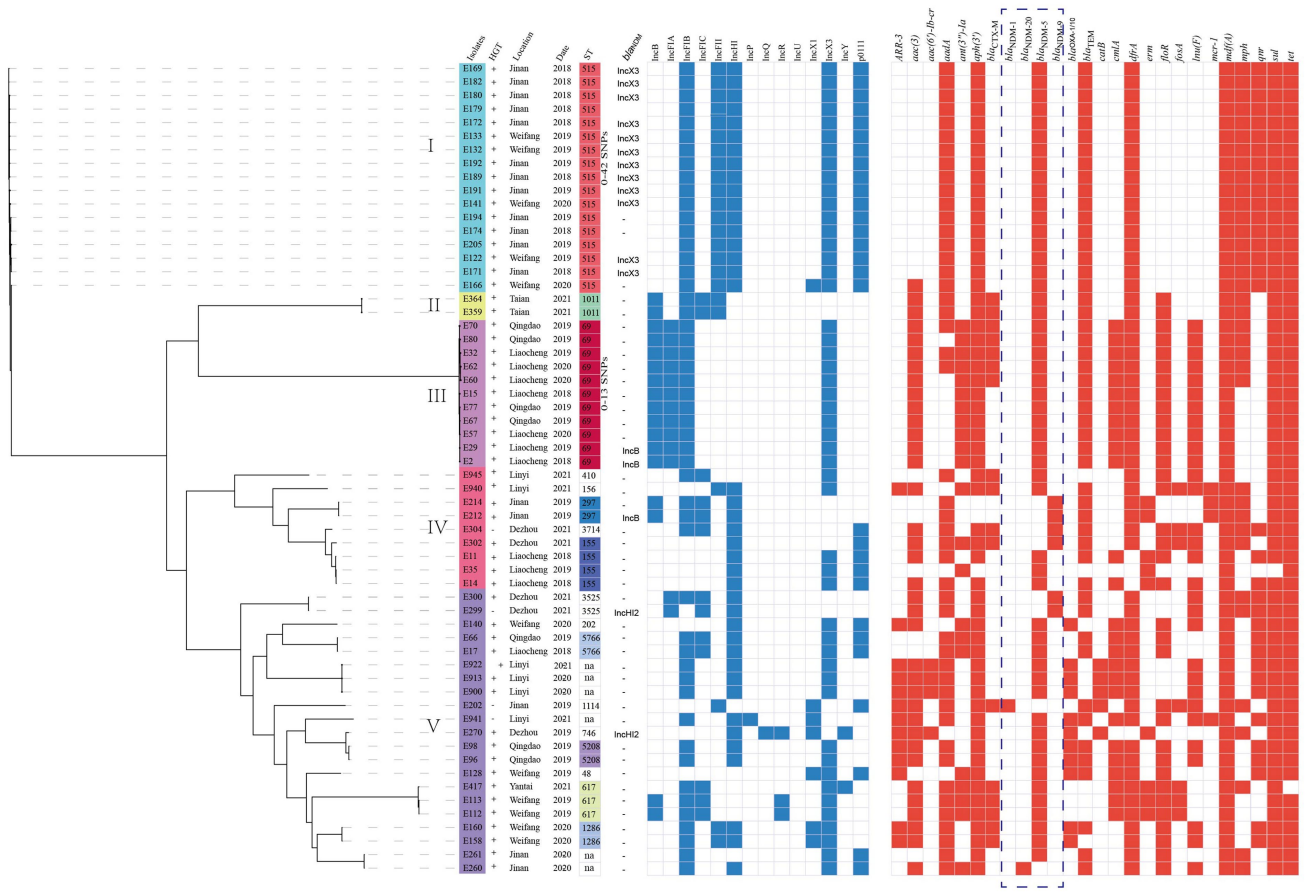
Phylogenetic analysis of NDM-positive *E. coli* isolates in this study (*n* = 60). Bayesian evolutionary tree was constructed using core-genome SNPs. Each isolate is labeled with the city of isolation year and ST. The red-filled squares indicate the possession of the indicated antimicrobial resistance genes (ARGs).

Whole-genome sequencing data were generated for 60 NDM-positive *E. coli* isolates, and the WGS results revealed that these isolates were categorized into 18 distinct STs, with 6 isolates classified as unclassified STs. Overall, ST515 (28.3%, 17/60) was the most prevalent isolate in Jinan and Weifang, followed by ST69 (18.3%, 11/60) in Qingdao and Liaocheng. These findings indicate a distinct geographic distribution preference (Figure 3). A phylogenetic tree was established using 60 NDM-positive *E. coli* isolates. Phylogenomic analysis revealed that all the *E. coli* isolates were classified into five distinct lineages. It is worth noting that all isolates in the lineages I belonged to ST515 and were sourced from Jinan and Weifang, with these isolates sharing only 0 ~ 24 core-genome SNPs (cgSNP) among themselves. In addition, isolates in lineage III belonged to ST69 and originated from Qingdao and Liaocheng, sharing only 0 ~ 13 SNPs among them (Figure 3). These findings indicated the clonal transmission of NDM-positive ST69 and ST515 *E. coli* isolates across various regions. Furthermore, our phylogenomic analysis demonstrated that the majority of NDM-positive *E. coli* isolates exhibited a significant degree of variation in core-genome sequences, suggesting a high genetic diversity of NDM-positive *E. coli* isolates from chicken at retail markets in Shandong Province, China.

**Figure 4:**
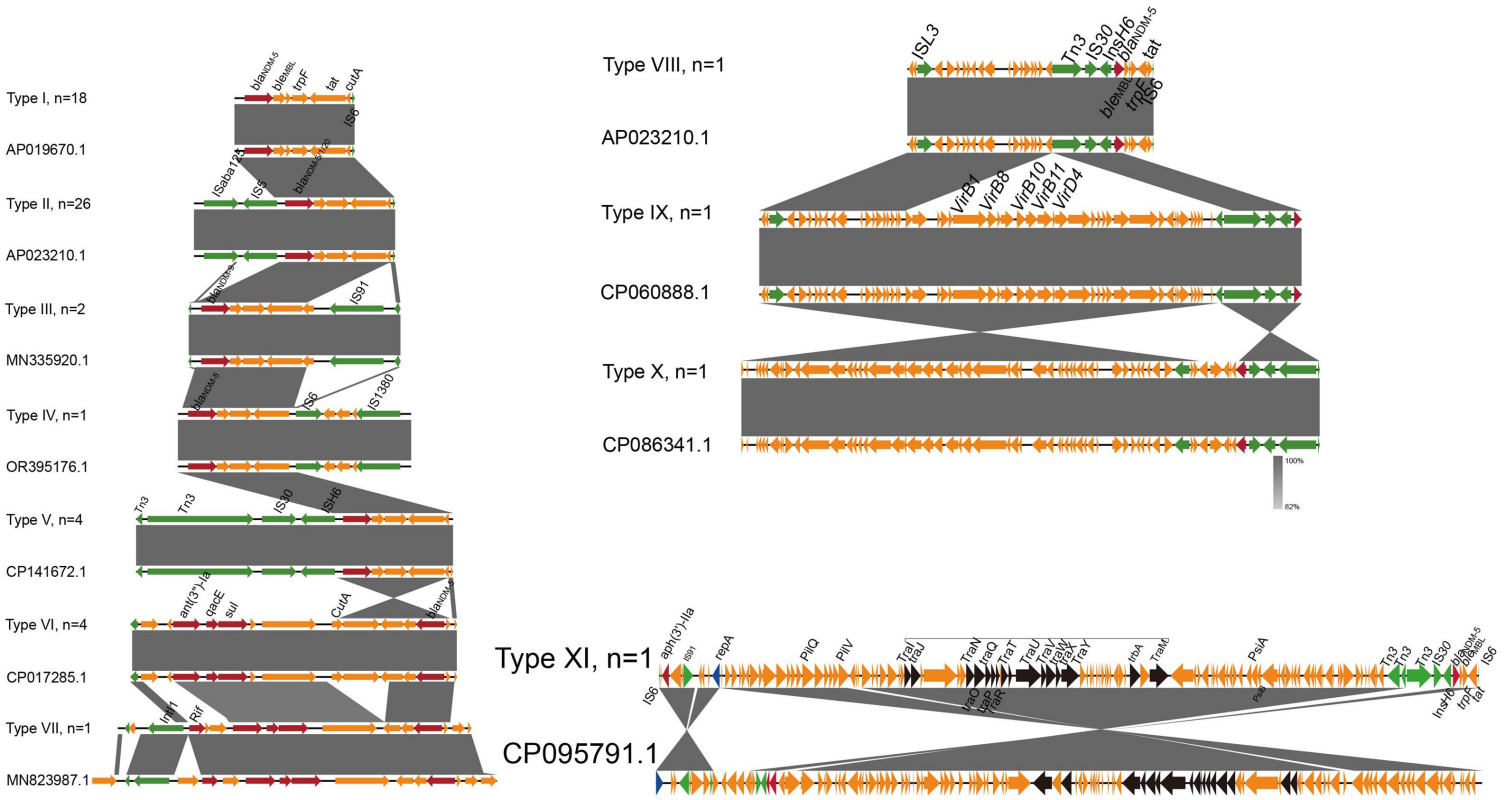
Phylogenetic structures of the NDM-positive *E. coli* isolates from this study and the GenBank database. The maximum likelihood tree shows the relationships among the 256 NDM-positive *E. coli* isolates. Isolate hosts from which isolates were obtained are indicated in the outer ring.

To further assess the relationship between the isolates from the current study and other resources in Shandong, China. A total of 196 NDM-positive *E. coli* isolates were collected in Shandong from the NCBI database (as of 2024). Then, a maximum likelihood phylogenetic tree was constructed using these 256 NDM-positive *E. coli* isolates. The 256 NDM-positive *E. coli* isolates were grouped into 8 clades and shared 216,099 core-genome SNPs (cgSNPs). It should be noted that 11 NDM-positive ST746 *E. coli* isolates from humans in Shandong (GenBank assembly GCA_001893215.1, GCA_001894425.1, GCA_001893995.1, GCA_001894025.1, GCA_001893225.1, GCA_001893375.1, GCA_001893305.1, GCA_001894035.1, GCA_001894065.1, GCA_001893765.1, and GCA_001894315.1) shared only 69–94 SNPs with NDM-positive ST746 *E. coli* isolates from a retail chicken in Shandong in this study (E270) (Figure 4), revealing a strong correlation of NDM-positive *E. coli* isolates among animals and foods.

### Plasmid analysis

A total of 14 incompatible group plasmid replicon types were detected among the 60 *bla*_NDM-_positive *E. coli* isolates including IncB (28%, 17/60), IncFIA (22%, 13/60), IncFIB (83%, 50/60), IncFIC (22%, 13/60), IncFII (38%, 23/60), IncX3 (82%, 49/60), IncHI (67%, 40/60), and other plasmids (Figure 3). Of note, WGS and BRIG analyses revealed that the *bla*_NDM_ genes may be located in IncX3 (*n* = 12), IncHI2 (*n* = 2), and IncB (*n* = 3) plasmids (Figure 3; Supplementary Figure S2). In addition, all of the IncX3 plasmid carrying *bla*_NDM_ genes were successfully transferred to recipients (*E. coli* C600^str^) by conjugation. These findings suggest that IncX3 plasmids may have undergone backbone fusion events with other plasmids, potentially driving horizontal gene transfer of resistance determinants.

### Analysis of antibiotic resistance genes

We conducted a comprehensive antimicrobial analysis on *E. coli* isolates that revealed the presence of the *β*-lactam resistance genes (*bla*_NDM-1_, *bla*_NDM-20_, *bla*_NDM-5_, *bla*_NDM-9_, *bla*_OXA-1/10_, *bla*_CTX-M_, and *bla*_TEM_). Other important resistance determinants that confer resistance to quinolones (*qnr*), aminoglycosides (*aadA*, *aph*, *armA* and *aac*), fosfomycin (*fosA*), chloramphenicol/florfenicol (*floR*), sulfonamides (*sul*), macrolide (*mdf(A)* and *mph(A)*), rifampicin (*ARR-3*), tetracycline (*tet*), and trimethoprim (*dfrA*). In addition, we identified two isolates (E212 and E214) that co-harbored *bla*_NDM-9_ and *mcr-1*, as well as two isolates (E940 and E941) that co-harbored *bla*_NDM-5_ and *mcr-1* (Figure 3).

### The genetic environments of *bla*_NDM_

**Figure 5:**
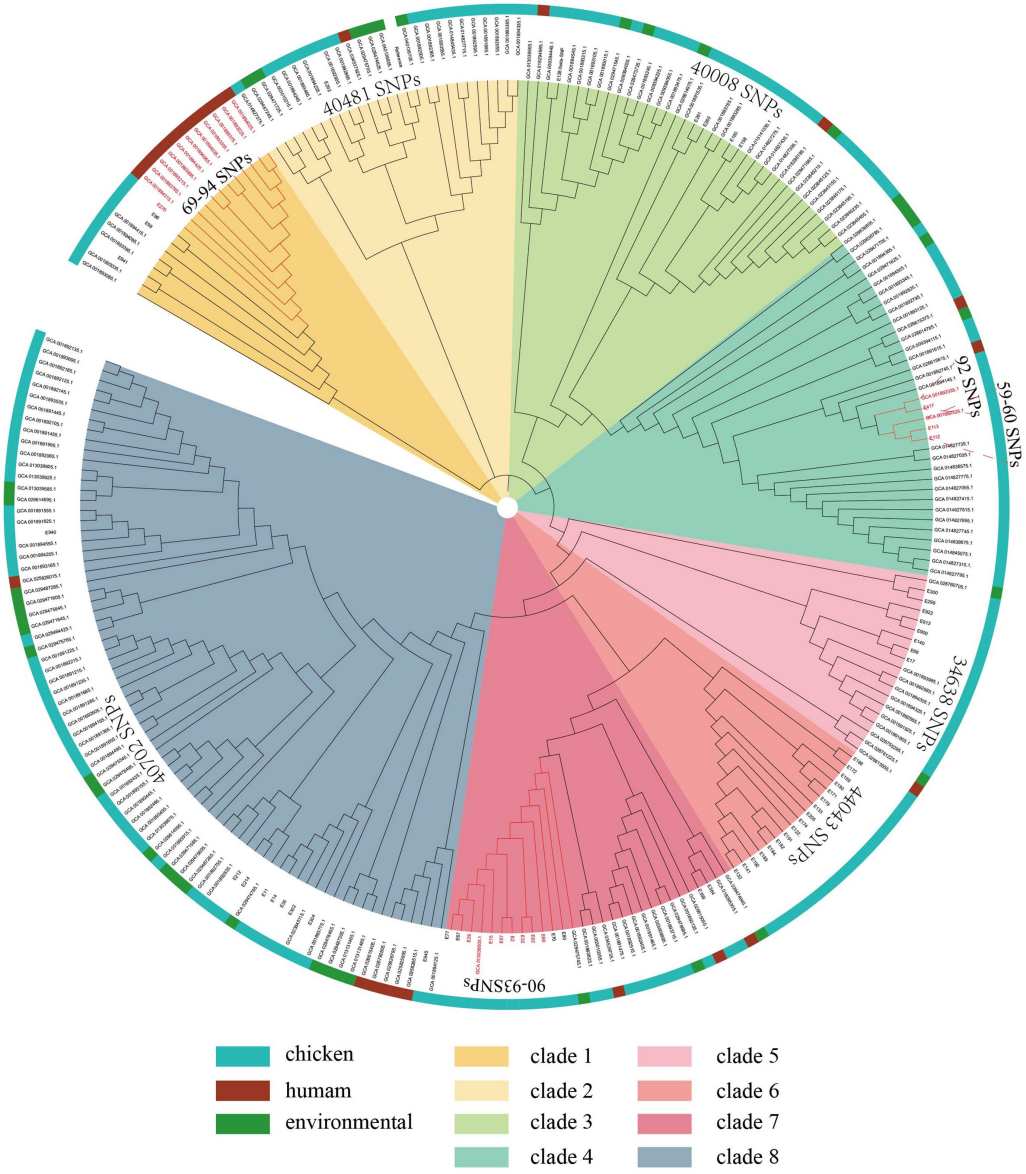
Genomic environments of *bla*_NDM_ of *E. coli* isolates. The figure was generated using Easyfig. Regions of homology are marked by shading, and regions of ≥ 99.0% nucleotide sequence identity are shaded gray. Arrows indicate the direction of transcription of the genes. In addition, red represents resistance gene, blue represents mobile genetic elements, black represents transfer gene tra clusters, and yellow represents other coding sequence (CDS).

A total of 11 genetic contexts (type I to type XI) were found in 60 isolates (Figure 5). All of the type VII and XI genetic contexts were novel in the GenBank database. Notably, *bla*_NDM-5_ was found in 10 of the 11 genetic context types. In all types, various variants of *bla*_NDM_ (*bla*_NDM-1_, *bla*_NDM-5_, *bla*_NDM-9_, and *bla*_NDM-20_) were directly associated with the *ble*_MBL_-*trpF*-*tat* genes. The type I arrangement was *bla*_NDM_-*ble*_MBL_-*trpF*-*tat*-*cutA* and was inserted upstream of In*sH6*. *bla*_NDM-1_ and *bla*_NDM-20_ were located in type II genetic contexts, which are identical to the type II context of *bla*_NDM-5_. In addition, we found that the type II genomic context IS*Aba125*-IS*5*-*bla*_NDM_-*ble*_MBL_-*trpF*-*tat*-IS*6* was the most prevalent NDM genetic environment in this study (43.3%, 26/60), which is identical to a *bla*_NDM-5_-carrying plasmid of carbapenem-resistant *Enterobacteriaceae* isolates from humans in China (Yang et al., 2022). Meanwhile, the backbone structure of *bla*_NDM-5_ including the IS*Aba125*-IS*5*-*bla*_NDM_-*ble*_MBL_-*trpF*-*tat*-IS*6* was usually carrying the IncX3-type plasmid and highly conserved. In the type VIII contexts, the IS*91* was inserted downstream of In*sH6*, and *bla*_NDM-9_ was uniquely associated with the single distinct genetic context. Compared with type VIII, the type IV context revealed deletion of IS*91*, but the IS*1380* was inserted upstream of In*6*. Type V contains the highest number of mobile genetic elements, including Tn*3*, IS*30*, and IS*H6*. In type VI, the *ant(3″)-Ia*, *qacE*, and *sul* resistance genes were located upstream of *bla*_NDM_. Type VII was similar to type III, with the exception that a *rif* resistance gene was found upstream of *ant(3″)-Ia*. In the type VIII contexts, the IS*L3* was inserted upstream of type V. In contrast, type IX inserts a virulence gene (*vir*) cluster at the Tn*3* of type VIII. The sequence of the type IX plasmid, when compared to the IncX3 plasmids (GenBank: CP060888.1), from an *E. coli* strain isolated from a patient in Zhejiang province, showed 100% nucleotide identity and 100% coverage (Yang et al., 2022). Based on type IV, type X reverses the upstream of *bla*_NDM_ with the action of IS*L3* and IS*H6*. Type XI reverses the upstream of *bla*_NDM_ through the action of Tn*3*, with the plasmid transfer gene *tra* cluster positioned upstream of *bla*_NDM_.

## Discussion

In the present study, we investigated the prevalence of NDM-positive *E. coli* isolates from chickens in the retail markets in Shandong, China, during 2018–2020. Recent studies have revealed that *bla*_NDM_ was the most frequently identified mobile carbapenem resistance gene found in various *Enterobacteriaceae* species, especially in Asia (Wu et al., 2023). However, NDM-positive *E. coli* isolates from retail chicken were rarely detected. In this study, we obtained 11.30% of NDM-positive *E. coli* isolates from retail chicken in markets in Shandong, China. Furthermore, our previous study also found that 15.42% of NDM-positive *E. coli* were highly represented in ducks in the coastal areas of China, which is similar to our result (Wang et al., 2021a,b). In contrast, the overall incidence of NDM-positive strains in animals was lower in pigs (6.05%) (Wen et al., 2023), suggesting that the *bla*_NDM_ gene is highly prevalent in poultry. Furthermore, a recent study found that the abattoir is a hotspot for cross-contamination, amplifying *bla*_NDM_ (Fu et al., 2024). The *bla*_NDM_ gradually increases along the chicken (4.70%)–slaughterhouse (7.60%)–retail (65.56%) chain (Fu et al., 2024), suggesting that NDM-positive *E. coli* reaches the dinner table via the farm–slaughterhouse–retail route, thereby endangering human health.

WGS analysis demonstrated the predominance of NDM-5 in this study. In fact, *bla*_NDM-5_ was first reported in an *E. coli* strain isolated from a patient in the UK in 2011 (Hornsey et al., 2011). Since then, *bla*_NDM-5_ has been detected in more than 40 countries, especially in China and Southeast Asia (Lv et al., 2022). Of further concern, NDM-5-producing *Enterobacterales* have been recovered from a variety of other sources worldwide, including food, livestock, companion animals, wildlife, and the environment (Hans et al., 2023). Plasmid profiling demonstrated that *bla*_NDM_ genes were predominantly harbored by IncX3-type plasmids, with significantly lower carriage rates observed in IncFII and IncHI plasmid types. In fact, IncX3 plasmids are the most common type of plasmid carrying *bla*_NDM-5_ in China (Ma et al., 2020). The IncX3 plasmids found in this study further highlight the importance of the epidemic IncX3 plasmid in the spread of the *bla*_NDM-5_ gene within the chicken market. MLST analysis revealed that these isolates belonged to 18 distinct STs, indicating a high diversity of STs in NDM-positive *E. coli* isolates from Chicken at retail markets. Our previous study found that the NDM-positive *E. coli* isolates from duck farms had 30 distinct STs and showed obvious distinctive diversities in geographical distribution (Wang et al., 2021a,b). In addition, it was also found that NDM-positive *E. coli* from raw meat in retail markets were classified into 14 different STs (Nisa et al., 2024); these studies showed that the ST diversity of NDM-positive *E. coli* from retail market chickens was lower than that from farms and higher than that from raw meat. In this study, ST69 and ST515 are the most prevalent *E. coli* isolates. Previous studies have also identified ST69 *E. coli* isolates as emerging and high-risk clones and were one of the most frequent clinical isolates in urinary tract infections (Cuénod et al., 2023; Mattioni Marchetti et al., 2023), while ST515 *E. coli* isolates have only been prevalent in vegetable markets in Northern Thailand (Chotinantakul et al., 2022). This study showed that there was cross-regional clonal transmission of ST69 between Liaocheng and Qingdao, and of ST515 between Jinan and Weifang. This pattern of cross-regional dissemination is particularly evident in the coastal areas of China and other developed regions, such as the grass carp were recovered from different markets and different sample booths in Guangdong, and the ducks were recovered from different farms in Anhui (Guo et al., 2024; Lv et al., 2022). In addition, NDM-positive ST69 and ST515 *E. coli* isolates persist in their host strain during 3 years of ongoing investigation, suggesting that NDM-positive ST69 and ST515 *E. coli* isolates may pose challenges in their treatment (Wang et al., 2018).

A total of 11 genetic contexts (types I to XIII) were found in 60 NDM-carrying plasmids. In type VI, a fusion plasmid was found that was recombined from an IncX3 plasmid carrying *bla*_NDM_ and an IncF plasmid carrying *ant(3ʹ)-Ia*, *qacE*, *sul*. This indicated that the formation of IncX3-FIB hybrid plasmids through the integration of IncF plasmids into IncX3 plasmid backbones probably facilitated the transmission of IncX3-F plasmids and ARGs (Bai et al., 2023). For type IX, it was confirmed that the *bla*_NDM_-carrying plasmid could mediate the transmission of the virulence plasmid through the formation of a fusion plasmid by Tn*3*. The mobile genetic element Tn*3* plays an important role in the transmission of the NDM gene (Li et al., 2024). These results indicated a diversity of genetic environments for*bla*_NDM_ in the Enterobacteriaceae.

WGS analysis also revealed that *bla*_NDM_ coexisted with other 30 other types of ARGs, 15 of these ARGs were highly prevalent with detection rates >50%. Previous research has shown that plasmid fusion can expand the host range of plasmids, accelerating the dissemination of ARGs among various bacteria (Lv et al., 2022), and can further promote the spread and persistence of carbapenem-resistant microbes in retail chicken at market. In this study, we first describe an *E. coli* isolate of coexistence of the *mcr-1* and *bla*_NDM_ genes. The co-occurrence of *bla*_NDM_ and *mcr-1* positive plasmids may have occurred, and several studies have been reported (Liu et al., 2024; Nisa et al., 2024). Of note, *mcr-1*—which confers resistance to the last-resort antibiotic colistin (Liu et al., 2023)—was detected in 4 NDM-positive *E. coli* isolates (2 *bla*_NDM-5_ and *mcr-1* and 2 *bla*_NDM-9_ and *mcr-1*). *bla*_NDM_ and *mcr-1* antimicrobial resistance genes confer resistance to colistin and carbapenems, which are antimicrobials often used as last-resort antibiotics in hospitals (Bai et al., 2023). Some recent studies have reported that *bla*_NDM-5_ and *mcr-1* co-producing *E. coli* isolates were recovered from food animals (Wang et al., 2021a,b). The results highlight that the food animals in markets serve as an important reservoir of *E. coli* isolates carrying *bla*_NDM_ and *mcr-1*, posing a serious public health threat via food chain transmission.

## Data Availability

The datasets presented in this study can be found in online repositories. The names of the repository/repositories and accession number(s) can be found in the article/Supplementary material.

## Footnotes

^1^ https://www.genomicepidemiology.org/services/

^2^ https://www.ncbi.nlm.nih.gov/pathogens
