# Supplementary material for: Molecular epidemiology of New Delhi metallo-β-lactamase-producing Escherichia coli in retail market chickens, Shandong, China
Source: Front Microbiol. 2025 Apr 22;16:1550742. doi: 10.3389/fmicb.2025.1550742 (PMC12052942; doi:10.3389/fmicb.2025.1550742)
Supplement: Supplementary file 1 [file Data_Sheet_1.pdf]

# Supplementary Material

## Supplementary Figures

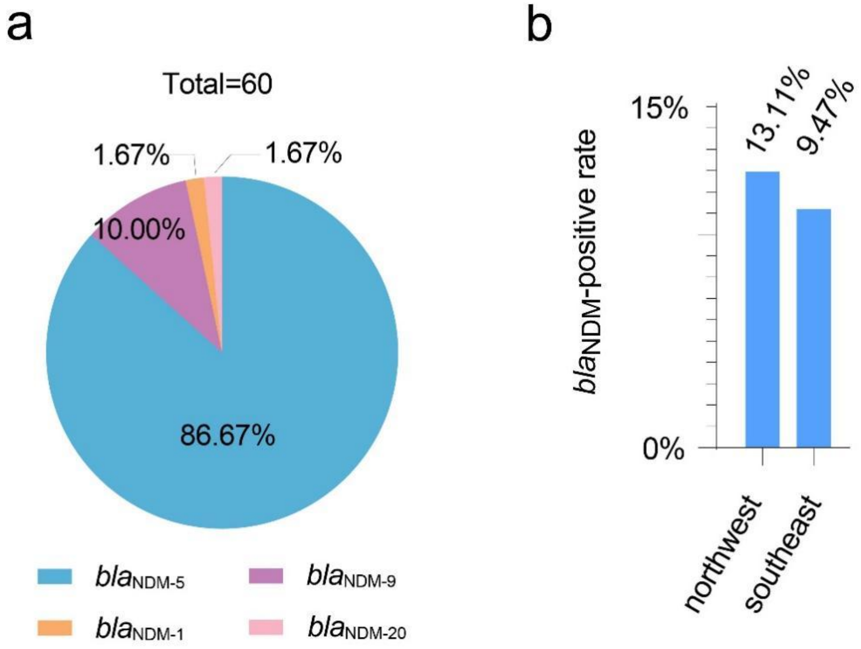


**Figure S1.** Distribution of the predominant variants *bla*$_{NDM}$ (a) and (b) *bla*$_{NDM}$ genes in different regions.

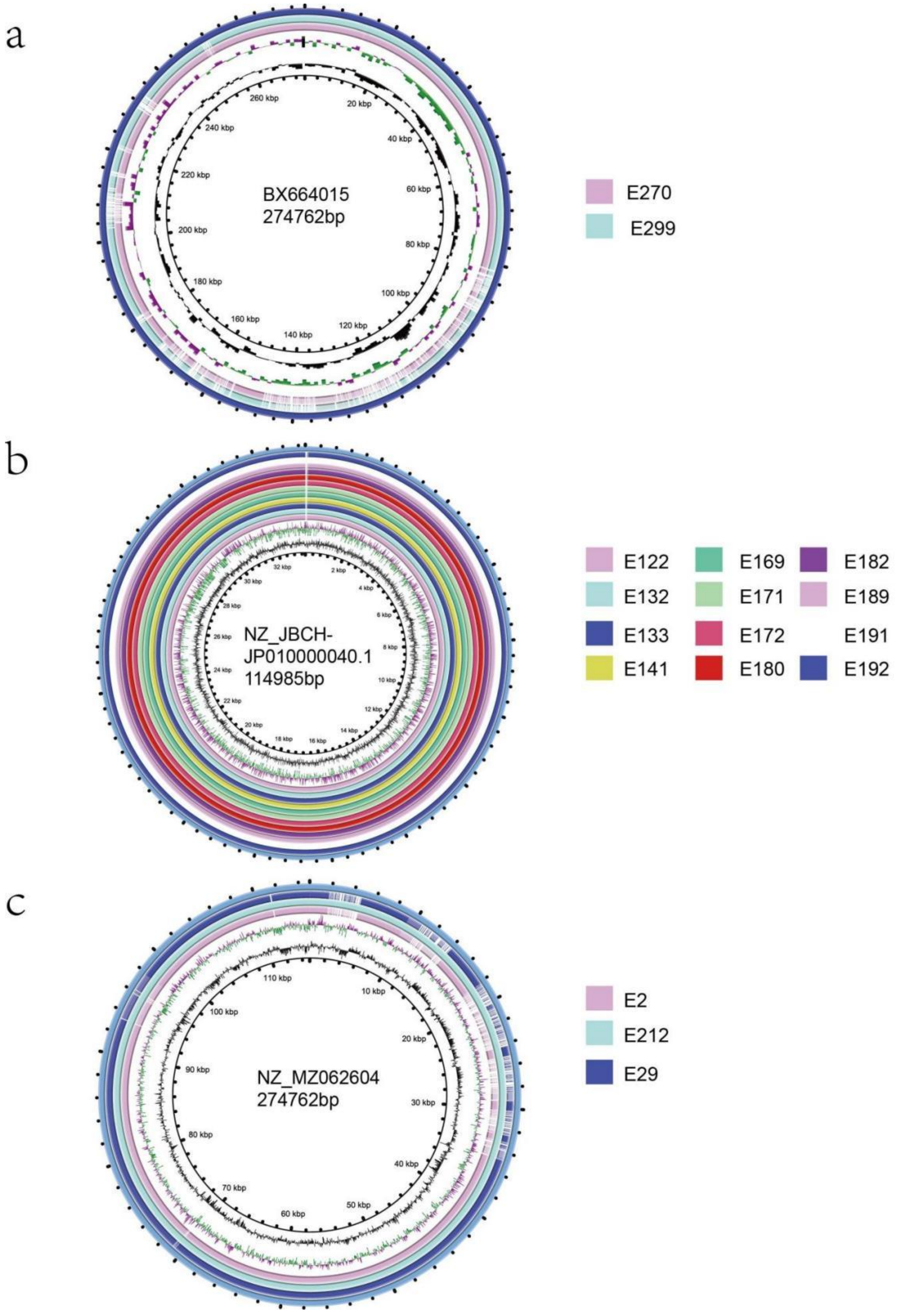


**Figure S2.** Sequence alignments of *bla*$_{NDM}$-bearing IncHI2 (a), IncX3 (b) and IncB (c) plasmids for long range MinION sequencing and all *E. coli* strains from this study. Genes depicted in the outer circle belong to BX664015, NZ_JBCHJP010000040.1 and NZ_MZ060604 included as a reference and the other circles represent structurally similar plasmids as indicated
